# Clinical T1a Renal Cell Carcinoma, Not Always a Harmless Disease—A National Register Study

**DOI:** 10.1016/j.euros.2022.03.005

**Published:** 2022-04-01

**Authors:** Tarik Almdalal, Pernilla Sundqvist, Ulrika Harmenberg, Mikael Hellström, Magnus Lindskog, Per Lindblad, Svan Lundstam, Börje Ljungberg

**Affiliations:** a Department of Surgery and Urology, Eskilstuna Country Hospital, Eskilstuna, Sweden; b Department of Urology, Faculty of Medicine and Health, Örebro University, Örebro, Sweden; c Department of Oncology, Karolinska University Hospital and Karolinska Institute, Stockholm, Sweden; d Department of Radiology, Sahlgrenska Academy/Sahlgrenska University Hospital, Gothenburg University, Gothenburg, Sweden; e Department of Immunology, Genetics and Pathology, Uppsala University, Uppsala, Sweden; f School of Medical Sciences, Faculty of Medicine and Health, Örebro University, Örebro, Sweden; g Department of Urology and Oncology, Sahlgrenska Academy, University of Gothenburg, Gothenburg, Sweden; h Department of Surgical and Perioperative Sciences, Urology and Andrology, Umeå University, Umeå, Sweden

**Keywords:** Radical nephrectomy, Partial nephrectomy, Renal cell carcinoma, T stage, Radiofrequency ablation, Cryoablation, Overall survival, Tumor size, Renal cell carcinoma type

## Abstract

**Background:**

T1a renal cell carcinoma (RCC) is typically considered a curable disease, irrespective of the choice of local treatment modality.

**Objective:**

To identify factors associated with the risk of local and distant recurrence, and overall survival (OS) in patients with primary nonmetastatic clinical T1a RCC.

**Design, setting, and participants:**

A population-based nationwide register study of all 1935 patients with cT1a RCC, diagnosed during 2005–2012, identified through The National Swedish Kidney Cancer Register, was conducted.

**Outcome measurements and statistical analysis:**

Outcome variables were recurrence (local or distant) and OS. Possible explanatory variables included tumor size, RCC type, T stage, surgical technique, age, and gender. Associations with disease recurrence and OS were evaluated by multivariable regression and Cox multivariate analyses, respectively.

**Results and limitations:**

Among 1935 patients, 938 were treated with radical nephrectomy, 738 with partial nephrectomy, and 169 with ablative treatments, while 90 patients had no surgery. Seventy-eight (4%) patients were upstaged to pT3. Local or metastatic recurrences occurred in 145 (7.5%) patients, significantly more often after ablation (17.8%). The risk of recurrence was associated with tumor size, upstaging, and ablation. Larger tumor size, disease recurrence, and older age adversely affected OS, whereas partial nephrectomy and chromophobe RCC (chRCC) were associated with improved survival. Limitations include register design and a lack of comorbidity or performance status data.

**Conclusions:**

Upstaging and recurrence occurred, respectively, in 4.0% and 7.5% of patients with nonmetastatic RCCs ≤4 cm. Tumor size upstaging and ablation were associated with the risk for recurrence, while tumor size and recurrence were associated with decreased OS. Patients with chRCC and partial nephrectomy had prolonged OS in a real-world setting.

**Patient summary:**

We studied factors that may influence the risk of disease recurrence and overall survival, in a large nationwide patient cohort having nonmetastatic renal cell carcinoma ≤4 cm. Tumor size, tumor type, and treatment were associated with the risk of recurrence and overall death. Partial nephrectomy prolonged overall survival.

## Take Home Message

Upstaging and disease recurrence were non-negligible in cT1a nonmetastatic renal cell carcinoma (RCC) patients in the real world. Tumor size, upstaging, and ablation were associated with recurrence risk, while tumor size and recurrence were associated with decreased overall survival (OS). Patients with chromophobe RCC and partial nephrectomy achieved prolonged OS.

## 1 Introduction

The majority of solid renal masses are malignant tumors, mostly renal cell carcinomas (RCCs) [1]. The proportion of incidentally diagnosed RCCs has increased during the last decades, likely due to an increased use of cross-sectional imaging for a variety of unrelated causes [2], [3]. The incidentally detected renal tumors have caused a stage shift in RCCs, with smaller tumors, with less advanced stage, and thus with a favorable prognosis [4], [5]. The high percentage of detected small renal tumors, including a substantial proportion with benign histology, has reinforced the interest in active surveillance and nephron-sparing treatments [1], [6]. As a group, small RCCs generally have been regarded as harmless [7]. However, for RCC, there remains a risk for local invasion and metastatic spread despite small tumor size [8]. Patients who subsequently develop metastases face a poor prognosis [8].

It is therefore highly important to evaluate the malignant potential of T1a RCCs to ensure that sufficient preoperative risk assessment is performed and that necessary treatments are given, but also to avoid overtreatment. It is further important to highlight possible risk factors for a more aggressive behavior. Upstaging from clinical T1a to pathological T3 at diagnosis has been shown to have a negative prognosis due to a higher risk for tumor recurrence and reduced survival [9].

The present study aimed to evaluate the occurrence of local recurrence and metachronous distant metastases in relation to the clinical and pathological factors and primary treatment of patients with nonmetastatic cT1a RCCs in a nationwide population of unselected RCC patients.

## 2 Patients and methods

### 2.1 Study design, setting, and participants

A total of 1935 patients, registered in the National Swedish Kidney Cancer Register (NSKCR) from January 2005 to the end of 2012 with clinical T1aM0 RCC at primary diagnosis, were included in the study. Patients with evidence of metastatic disease at diagnosis were excluded. Patients with cT1aM0 RCC were linked to the Swedish National Population Register for overall survival (OS) information. Information about the patient’s primary treatments, tumor-node-metastasis (TNM) stage, RCC type, Fuhrman grade, tumor size, and patient’s age and sex were extracted from the NSKCR. The TNM classification system and Fuhrman grade classification was used [10], [11]. Seventeen patients registered as having Tx were reclassified to have T1a, having similar clinical behavior to T1a RCCs. Tumor size was defined as the maximal tumor diameter measured by computerized tomography (CT) or magnetic resonance imaging (MRI). All patients had potentially at least a 5-yr follow-up time for disease recurrence. Follow-up OS data were available for all 1935 patients until December 2019. At that time, 1429 (73.9%) patients were alive and 506 (26.1%) had died of any cause.

### 2.2 Statistical analyses and outcome measures

Outcome variables were recurrent disease (local or distant) and OS. OS time was defined as the time from diagnosis to the date of death from any cause or alive at the end of 2018.

Possible explanatory variables included age, gender, tumor size, RCC type (clear cell RCC [ccRCC], papillary RCC [pRCC], chromophobe RCC [chRCC], or other), and surgical technique (radical nephrectomy [RN], partial nephrectomy [PN], ablative treatment, or other). The category ablative treatment was a composite that included radiofrequency ablation (*n* = 142), cryoablation (*n* = 25), and high-intensity focused ultrasound (*n* = 2).

### 2.3 Selection bias

The NSKCR has a coverage of 99% of all RCCs diagnosed, compared with the Swedish Cancer Register where all patients with any cancer are reported according to the Swedish law. All Swedish citizens have a mandatory unique personal identity number that is used for the registration and follow-up on OS [12]. For the data presented in this paper, 15% of the patients’ charts (291 of 1935) were validated by NSKCR panel members. All patients primarily registered with a tumor size of <10 mm were intentionally validated. Mismatched data were revalidated and corrected for this analysis. The entire register has furthermore been validated showing high quality of the NSKCR data [13].

### 2.4 Statistical methods and missing data

The Mann-Whitney U test was used to compare medians and means of independent groups, while chi-square tests were performed for comparison of proportions. OS was estimated by the Kaplan-Meyer method and analyzed by the log-rank test. Multivariable analyses using logistic regression and Cox regression models were performed to identify potential prognostically independent variables. A two-tailed *p* value of <0.05 was considered statistically significant. Statistical analysis was performed using the SPSS version 22.

### 2.5 Ethical approval

The present study was approved by the regional Ethical Review Board of Northern Sweden (Dnr 2012-418-31M).

## 3 Results

Among the 1935 patients, 1192 (61.6%) were males and 743 (38.4%) females. Their mean age was 64.3 yr, ranging from 19 to 91 yr. Males were younger than females at diagnosis (mean 63.6 vs 65.3 yr, *p* = 0.002). Patients treated with PN were significantly younger than the other treatment groups (*p* < 0.001 for all), as shown in Table 1, and patients treated with RN were younger than patients treated with ablation (*p* = 0.038). Most (86.9%) patients were treated surgically, 169 (8.7%) with ablation, and 84 (4.3%) were treated nonsurgically, as shown in Table 1.

**Table 1:** Distribution of patient’s characteristics shown in relation to type of treatment in 1935 patients with cT1aM0 RCC

Variable		Radical nephrectomy (*n* = 938)	Partial nephrectomy (*n* = 738)	Ablation treatment (*n* = 169)	Other (*n* = 90)	All (*n* = 1935)
Age (yr)	Mean a	65.7	60.6	67.3	73.6	64.3
	Median (range)	67 (26–88)	62 (19–85)	68 (20–85)	75 (31–91)	66 (19–91)
Tumor size	Mean b	32.4	27.5	24.9	29.4	29.7
	Median (range)	35 (6–40)	27 (10–40)	25 (10–40)	30 (3–40)	30 (3–40)
Gender	Men	572	460	106	54	1192
	Women	366 (39.0%)	278 (37.7%)	63 (37.3%)	36 (40.0%)	743 (38.4%)
T stage	pT1a	872 c	727	169	89	1857
	pT3a	66 (7.0%)	11 (1.5%)	0	1 (1.1%)	78 (4.0%)
RCC type	ccRCC	746	525	114	34	1419
	pRCC	124	155	29	10	318
	chRCC	54	50	9	2	115
	Other/unknown	14	8	17	42	80
Recurrent disease	No	874	694	139 d	83	1790
	Yes	64 (6.8%)	44 (6.0%)	30 (17.8%)	7 (7.8%)	145 (7.5%)
Overall survival	Alive	637 e	629 e	127	36	1429
	Dead	301 (32.1%)	109 (14.8%)	42 (24.9%)	54 (60.0%)	506 (26.1%)

ccRCC = clear cell RCC; chRCC = chromophobe RCC; HIFU = high-intensity focused ultrasound; pRCC = papillary RCC; RCC = renal cell carcinoma.

Percentage is counted as gender, number of patients with upstaging to pT3 upstage, disease recurrence, and overall survival status in relation to the total number of comparable patients.

Ablation included radiofrequency ablation (*n* = 142), cryoablation (*n* = 25), and HIFU (*n* = 2). Other included eight patients with other surgeries and 82 without surgery.

a Patients with partial nephrectomy (PN) were younger than in the other treatment groups (*p* < 0.001 for all), patients with ablation were older than patients treated with radical nephrectomy (RN; *p* = 0.038).

b All treatment groups differed significantly in tumor size between each other (*p* < 0.001 for all except between other vs RN and PN, *p* = 0.001 and *p* = 0.12, respectively).

c RN had pT3 stage more often than PN (*p* < 0.001).

d Patients treated with ablation had recurrent disease significantly more often (*p* < 0.001).

e Patients treated with RN had significantly higher overall death rate than patients treated with PN (*p* < 0.001).

There were 73.3% patients with ccRCC, 16.4% with pRCC, and 5.9% with chRCC, and 1.7% had unclassified RCC types. Fifty-one (2.6%) patients were registered with unknown histology. Among these, 39 were managed by nonsurgical treatments, ten with ablation, and one with RN, and one was registered with other surgery. Of all patients with cT1a, 4.0% were upstaged to pT3 (Table 1). Most cT1a RCCs upstaged to pT3 (75 of 78, 96%) had tumor size between 20 and ≤40 mm, while three of 78 (4%) upstaged cT1a had a tumor size of ≤2 cm (*p* < 0.001).

At follow-up, 145 (7.5%) patients were diagnosed with local recurrence or distant recurrent disease (Table 1). Local recurrence in the treated kidney was found in 45 patients, and seven patients had true renal fossa recurrences. The most common distant recurrence sites were the lung found in 51 patients, bone in 16, lymph nodes in 15, contralateral kidney in 12, liver in 12, and brain in five (Table 2). There was no difference in recurrence rate between males and females (8.2% vs 6.7%, *p* = 0.232). In a univariable analysis, patients older than 65 yr at diagnosis had recurrent disease more frequently than younger patients (8.6% vs 6.2%, *p* = 0.046). Twenty of 78 patients upstaged to pT3 (25.6%) developed recurrent disease, significantly more frequently than 125 of 1857 patients with pT1a (6.7%, *p* < 0.001). Recurrent disease was diagnosed in 15 patients with tumor size ≤2 cm compared with 130 patients with recurrence with tumor size varying from 2 to ≤4 cm (*p* = 0.006).

**Table 2:** Distribution of sites of disease recurrence in relation to histological tumor type in 145 patients among the 1935 patients with cT1aM0 renal cell carcinoma at primary diagnosis

Site of recurrence a	ccRCC (*n* = 1419)	pRCC (*n* = 318)	chRCC (*n* = 115)	Other/unknown (*n* = 83)	All a (*n* = 1935)
All	138	32	6	12	188
Lung	39	7	2	3	51
Treated kidney	27 (23 b)	11 (9 b)	3 (2 b)	4 (2 b)	45 (36 b)
Skeletal	12	2	0	2	16
Lymph nodes	12	2	9	1	15
Adrenals	9	1	0	0	10
Contralateral kidney	9	3	0	0	12
Liver	9	2	0	1	12
Adrenals	9	1	0	0	10
Local groin	4	2	0	1	7
Brain	4	1	0	0	5
Other	13	1	1	0	15
Patients with recurrences	109	24	5	7	145

ccRCC = clear cell renal cell carcinoma; chRCC = chromophobe renal cell carcinoma; pRCC = papillary renal cell carcinoma.

a Patients may have more than one site of recurrence: 33 patients had two recurrence sites, eight patients had three sites, and two patients had four sites registered.

b Patients with recurrent disease in the treated kidney only (when recurrences are subdivided into local recurrence in the treated kidney only).

The recurrence rate was 7.7% for patients with ccRCC, 7.5% for pRCC patients, and 4.3% for patients with chRCC (Table 2). There was no difference in disease recurrences between patients treated with RN and PN (6.8% and 6.0%, respectively), while patients treated with ablative techniques had a higher rate (30 of 169, 17.8%) than patients treated with RN or PN (*p* < 0.001). Of 30 ablative patients with recurrences, 22 developed local recurrence in the treated kidney only, while eight developed distant recurrent disease. Recurrence in the treated kidney only was registered in 12 PN patients (1.6%), significantly less frequent than after ablation (*p* < 0.001). There was no statistical difference in the occurrence of distant metastases in patients treated with ablation versus those treated with PN (*p* = 0.584, data not shown).

In a multivariable analysis, tumor size, type of treatment, and pT stage were associated with an increased risk for recurrent disease, while gender, age, and RCC type were not (Table 3). Tumor size had a hazard ratio (HR) of 1.05 (95% confidence interval [CI] 1.03–1.09), while patients upgraded to pT3 RCCs had an HR of 5.37 (95% CI 3.00–9.59). Patients treated with ablation had a five-fold elevated risk for recurrence (HR 5.62, 95% CI 3.23–9.95; Table 3).

**Table 3:** Results of multivariate logistic regression analysis of factors important for the occurrence of recurrent disease in 1935 patients with nonmetastatic cT1 RCC

	HR	95.0% CI for HR		*p* value
		Lower	Upper	
Age (yr)	1.010	0.994	1.030	0.255
Gender (man vs woman)	0.797	0.540	1.178	0.207
Tumor size (mm)	1.053	1.029	1.089	<0.001
pT stage				
pT1a	Ref.			
pT3a	5.365	3.002	9.590	<0.001
Treatment				
Radical nephrectomy	Ref.			
Partial nephrectomy	1.417	0.911	2.203	0.122
Ablation	5.672	3.232	9.953	<0.001
RCC type				
ccRCC	Ref.			
pRCC	0.926	0.564	1.521	0.761
chRCC	0.425	0.150	1.208	0.108

CI = confidence interval; ccRCC = clear cell RCC; chRCC = chromophobe RCC; HR = hazard ratio; pRCC = papillary RCC; RCC = renal cell carcinoma; Ref. = reference.

Patients (*n* = 129) treated with surveillance, other surgeries, and/or other and unknown RCC types were excluded from the analysis for clarity.

**Fig. 1:**
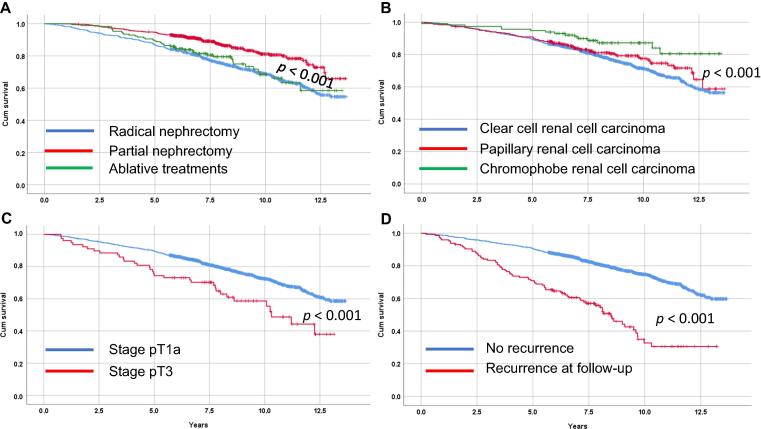
Kaplan-Meier curves of univariate survival probability for (A) the different treatments, (B) different RCC types, (C) stage pT1a or stage pT3, and (D) occurrence of recurrent disease or no recurrence. Cum = cumulative; RCC = renal cell carcinoma.

At the time of data analysis, 74.6% (1385 of 1857) of patients with pT1a RCCs were alive compared with 56.4% (44 of 78) of patients with pT3 (*p* < 0.001). In the univariable analysis, age, type of treatment, T stage, RCC type, tumor size, and recurrent disease were associated significantly with OS, as exemplified in Fig. 1A–D. Using a Cox multivariate analysis, age, tumor size, type of treatment, and recurrent disease all were associated negatively with OS, while patients treated with nephron sparing and patients with chRCC had better OS (Table 4). Age was significantly linked to OS with an HR of 1.076 (95% CI 1.064–1.088) per year of age. In addition, tumor size was associated independently with an increased risk (HR 1.017, 95% CI 1.003–1.032, *p* = 0.019). Patients diagnosed with recurrent disease had a three-fold higher risk for overall death (*p* < 0.001). When subdividing recurrences into local recurrence in the treated kidney only and distant recurrent disease, no OS association was observed in patients with local recurrence (HR 0.944, 95% CI 0.376–2.372), while risk for reduced OS increased to 3.8 in patients with distant recurrences (HR 3.782, 95% CI 2.862–4.997). Using RN as a reference, patients treated with PN had 23% better OS in the multivariate analysis (HR 0.771, 95% CI 0.609–0.976), while ablation lacked an independent effect (Table 4). A 49% reduced risk for death was observed for patients with chRCC with an HR of 0.513 (95% CI 0.308–0.861, *p* = 0.011) compared with ccRCC, while there was no difference in OS between patients with ccRCC and those with pRCC (Table 4).

**Table 4:** Results from Cox regression analysis of factors important for overall survival in 1935 patients with nonmetastatic cT1a RCC

	Unadjusted				Adjusted			
	HR	95% CI for HR		*p* value	HR	95% CI for HR		*p* value
		Lower	Upper			Lower	Upper	
Age (yr)	1.083	1.072	1.094	<0.001	1.076	1.064	1.088	<0.001
Gender	0.936	0.781	1.121	0.472	0.918	0.754	1.118	0.396
RCC type								
ccRCC	Ref.				Ref.			
pRCC	0.827	0.639	1.071	0.150	0.827	0.632	1.082	0.165
chRCC	0.469	0.280	0.786	0.004	0.513	0.306	0.861	0.011
pT stage								
pT1a	Ref.				Ref.			
pT3	1.920	1.356	2.719	<0.001	1.301	0.901	1.879	0.160
Treatment								
Radical nephrectomy	Ref.				Ref.			
Partial nephrectomy	0.513	0.412	0.640	<0.001	0.771	0.609	0.976	0.031
Ablation	0.917	0.664	1.268	0.602	0.731	0.502	1.065	0.103
Tumor size (mm)	1.043	1.031	1.056	<0.001	1.017	1.003	1.032	0.019
Recurrence	3.088	2.417	3.946	<0.001	3.185	2.433	4.169	<0.001

CI = confidence interval; ccRCC = clear cell RCC; chRCC = chromophobe RCC; HR = hazard ratio; other = other RCC types; pRCC = papillary RCC; RCC = renal cell carcinoma; Ref. = reference.

In total, 129 patients were excluded from the analysis for clarity due to other and unknown RCC types, and other and nonsurgical treatments.

## 4 Discussion

This register study, based on all patients with RCC nationwide, shows that a non-negligible proportion of nonmetastatic RCCs ≤4 cm were upstaged to locally aggressive tumors or developed recurrence within 5 yr of follow-up. Small renal masses <4 cm in size have generally been regarded as indolent and equally treatable with nephron-sparing surgery, ablation, or surveillance [1]. This attitude is supported by the findings in many studies where 25–30% of small renal masses turn out to have a benign histology [14]. However, in the present study on nonmetastatic cT1a RCC, around 4% of the patients were upstaged to pT3, having renal vein and/or perinephric fat invasion, and in addition, 7.5% of the patients were diagnosed with distant recurrent disease within the 5-yr follow-up time.

We showed that the risk of upstaging from cT1a to pT3 increased significantly by tumor size, in line with previous studies. Tan et al [15] reported 6.8% and Lee et al [16] reported 3.7% with pT3 upstage after a histopathological examination. The association between tumor size and upstaging to stage pT3 RCC was previously shown in an NSKCR study on cT1 RCCs [17] and in a recent meta-analysis [18]. Our present findings corroborate these reports, adding clinically important value by demonstrating that patients with upstaging have a significantly higher incidence of recurrent disease than those with pT1a RCC.

In the present study, we further found that a substantial proportion of patients (7.5%) were diagnosed with recurrent disease within a 5-yr follow-up time. The risk of recurrence was associated significantly with tumor size, with a 5% increase in the risk for each millimeter increase in size. Recently, Tan et al [15] published a single-center study of 565 patients with RCCs ≤4.0 cm, showing a 6% tumor recurrence rate, associated with tumor size, presence of symptoms, and age above 65 yr. In our study representing a larger nationwide cohort including RCC patients only, we confirm the association of recurrence risk with tumor size, but not with age. Furthermore, we showed that the choice of treatment affected the risk of disease recurrence. Patients treated with ablative therapy had a 5.7 time higher risk for recurrence as compared with those treated with RN, in line with previous studies [19]. Albeit most recurrences after ablative treatment were local in the treated kidney, the proportion of patients with distant recurrent disease was not statistically different from that of surgically treated patients. Patients treated with PN did not have a higher risk of recurrence than patients treated with RN. Our findings indicate that patients treated with ablative techniques need a more frequent follow-up schedule than those treated with PN or RN.

In our study, RCC type was not associated with the risk of recurrence either in the univariate or in the multivariable analysis. While patients with ccRCC and pRCC had an equal proportion of recurrences, patients with chRCC had a nonsignificant trend for fewer recurrences. In a study of Tan et al [15], recurrent disease was observed in 6–8% of patients with ccRCCs and pRCCs, while none of 11 chRCC patients had any recurrence. Lee et al [16] found even lower recurrent rates: 3% for ccRCCs and 1.5% for pRCC, while none of 120 patients with chRCC got recurrent disease.

Women were significantly older than men in our study, in line with previous findings [20], [21]. The reason for this difference in age distribution between genders is unclear. We found that females were treated with PN equally to men (38.6% vs 37.4%), in contrast to the findings of Metcalf et al [22], who found that gender differences persist in the management of cT1a RCC. In addition, Hadjipavlou et al [23] found that PN was associated with younger age, males, and smaller tumors. Neither age nor gender had any impact on the risk of disease recurrence in the present study.

Expectedly, an increase in age was significantly associated with OS, while gender was not. Patients treated with PN had significantly better OS than those treated with RN, as tested in a multivariate analysis adjusted for other potentially confounders. The OS advantage was expected in patients treated with PN, who were younger and possibly fitter than RN patients. However, the opposite applies for patients treated with ablation. These patients are generally older and have comorbidities more frequently, but these patients had no different OS from those treated with RN. Our finding with an OS advantage of the treatment with PN compared with RN opposes that of the only published randomized clinical study that found no survival benefit of PN [24]. However, that study was prematurely closed due to low inclusion, and only few patients were included yearly from each center, over a long time. It is well known that reduced renal function is associated with an increase in cardiovascular events and reduced OS [25]. Nephron-sparing approaches are supported in several nonrandomized studies [26], [27], [28]. The obvious advantage of nephron sparing is the preservation of renal function, while it also offers acceptable surgical morbidity, equivalent cancer control, and potential for better long-term survival [29].

Surprisingly, the univariate statistical significance of upstaging of cT1a to pT3 for OS did not remain significant when adjusted in the Cox analysis. In contrast, tumor recurrence, of any kind, was associated with a three-fold increase in the likelihood of death in the multivariable survival analysis. When subdivided, local recurrence in the treated kidney was not associated with a decreased OS risk, albeit having a broad CI. RCC type also influenced OS, with chRCC being an independent predictor of better survival. These results confirm previous studies on the prognostic impact of RCC type [30].

This register-based study has several important limitations. Clinical, radiological, and histopathological information was gathered from all Swedish hospitals, and chart data can be subjected to reporting errors. Moreover, the NSKCR did not contain information on performance status, comorbidities, pre- and postoperative complications, and cancer-specific survival. All patients, evaluated with CT or MRI at primary diagnosis, were treated based on patients’ and surgeons’ preferences. The strength of this material is that it represents real-world data of an unselected nationwide patient population with a 99% inclusion rate of newly diagnosed RCC in Sweden, which minimizes any selection bias, found in most other studies [31]. The long-term follow-up data and the highly valid data on OS strengthen the results obtained.

## 5 Conclusions

Patients with RCCs ≤4 cm without metastases at diagnosis have non-negligible risks of tumor upstaging and disease recurrence (4.0% and 7.5%, respectively). Tumor size, pathological upstaging, and ablative therapy were associated with disease recurrence. Furthermore, tumor size and disease recurrence were significantly associated with decreased OS, while patients with chRCC and those treated with PN were associated with improved OS.

***  Author contributions*:** Börje Ljungberg had full access to all the data in the study and takes responsibility for the integrity of the data and the accuracy of the data analysis.

*Study concept and design:* Ljungberg, Sundqvist.

*Acquisition of data:* Almadalal, Sundqvist, Harmenberg, Hellström, Lindblad, Lindskog, Lundstam, Ljungberg.

*Analysis and interpretation of data:* Almadalal, Ljungberg.

*Drafting of the manuscript:* Almadalal, Sundqvist, Ljungberg.

*Critical revision of the manuscript for important intellectual content:* Almadalal, Sundqvist, Harmenberg, Hellström, Lindblad, Lindskog, Lundstam, Ljungberg.

*Statistical analysis:* Almadalal, Ljungberg.

*Obtaining funding:* Almadalal.

*Administrative, technical, or material support:* Almadalal, Ljungberg.

*Supervision:* Ljungberg, Sundqvist.

*Other:* None.

***  Financial disclosures:*** Börje Ljungberg certifies that all conflicts of interest, including specific financial interests and relationships and affiliations relevant to the subject matter or materials discussed in the manuscript (eg, employment/affiliation, grants or funding, consultancies, honoraria, stock ownership or options, expert testimony, royalties, or patents filed, received, or pending), are the following: Tarik Almadalal and Pernilla Sundqvist: no conflict of interest. Ulrika Harmenberg: company speaker honorarium—Pfizer, BMS, and Ipsen; advisory boards—BMS and Ipsen. Mikael Hellström and Per Lindblad: no conflict of interest. Magnus Lindskog: advisory boards—BMS and Ipsen. Sven Lundstam: advisory boards—MSD, BMS, and Ipsen. Börje Ljungberg: company speaker honorarium—Novartis, Pfizer, Ipsen, and BMS; trial participation—Janssen, Astellas, and Medivation; company consultant—Janssen, Ipsen, and MSD; other—EAU.

***  Funding/Support and role of the sponsor*:** The study was supported by the Swedish Association of Local Authorities and Regions (SALAR) and the Clinical Research Center in the county of Sörmland, Sweden.

***  Acknowledgments*:** This project was made possible by the continuous work of the NSKCR of Sweden steering group: Börje Ljungberg (chairman), Peter Elfving, Marcus Thomasson, Britt-Inger Kröger Dahlin, Annika Håkansson, Åsa Jellvert, Pernilla Sundqvist, Per Lindblad, Magnus Lindskog, Ulrika Harmenberg, Ann-Helén Scherman-Plogell, Anders Kjellman, Andreas Thorstenson, Per Skoglund, Linn Pettersson, Emma Ulvskog, Magnus Fovaeus, Sven Lundstam, Mikael Hellström, Jörgen Jehander, and Martin Johansson. The authors also thank Soheila Hosseinnia and other collaborators at the Regional Cancer Centre, Stockholm/Gotland, for continuous work with the register and providing data from the NSKCR.
